# Single-Center Comparison of [^64^Cu]-DOTAGA-PSMA and [^18^F]-PSMA PET–CT for Imaging Prostate Cancer

**DOI:** 10.3390/curroncol28050353

**Published:** 2021-10-15

**Authors:** Siroos Mirzaei, Rainer Lipp, Shahin Zandieh, Asha Leisser

**Affiliations:** 1 Department of Nuclear Medicine with PET-Center, Clinic Ottakring (Wilhelminenspital), 1160 Vienna, Austria; AshaLeisser@gesundheitsverbund.at; 2 Department of Internal Medicine, Medical University of Graz, 8036 Graz, Austria; RainerLipp@gesundheitsverbund.at; 3 Department of Radiology and Nuclear Medicine, Hanusch Hospital, 1160 Vienna, Austria; Shahin.Zandieh@gesundheitsverbund.at

**Keywords:** [^64^Cu]/[^18^F] PSMA, oncology, prostate cancer, PSMA positron emission, tomography/computed tomography (PET–CT), prostate-specific membrane, antigen (PSMA)

## Abstract

Introduction: the diagnostic performance of [^64^Cu]-DOTAGA-PSMA PET–CT imaging was compared retrospectively to [^18^F]-PSMA PET–CT in prostate cancer patients with recurrent disease and in the primary staging of selected patients with advanced local and possible metastatic disease. Methods: We retrospectively selected a total of 100 patients, who were consecutively examined in our department, with biochemical recurrence after radical prostatectomy or who had progressive local and possible metastatic disease in the last 3 months prior to this investigation. All patients were examined with a dedicated PET–CT scanner (Biograph; Siemens Healthineers). A total of 250 MBq (3.5 MBq per kg bodyweight, range 230–290 MBq) of [^64^Cu]-DOTAGA-PSMA or [^18^-F]-PSMA was applied intravenously. PET images were performed 1 h post-injection (skull base to mid-thigh). The maximum standardized uptake values (SUVmax) of PSMA-positive lesions and the mean standardized uptake value (SUVmean) of the right liver lobe were measured. Results: All but 9/50 of the patients (18%; PSA range: 0.01–0.7 µg/L) studied with [^64^Cu]-DOTAGA-PSMA and 6/50 of the ones (12%; PSA range: 0.01–4.2) studied with [^18^F]-PSMA had at least one positive PSMA lesion shown by PET–CT. The total number of lesions was higher with [^64^Cu]-DOTAGA-PSMA (209 vs. 191); however, the median number of lesions was one for [^64^Cu]-DOTAGA-PSMA and two for [^18^F]-PSMA. Interestingly, the median SUVmean of the right liver lobe was slightly higher for [^18^F]-PSMA (11.8 vs. 8.9). Conclusions: [^64^Cu]-DOTAGA-PSMA and [^18^F]-PSMA have comparable detection rates for the assessment of residual disease in patients with recurrent or primary progressive prostate cancer. The uptake in the liver is moderately different, and therefore at least the SUVs of the lesions in both studies would not be comparable.

## 1. Introduction

Prostate cancer (PCa) is one of the most commonly diagnosed malignancies in men, with a total of 1,414,259 new cases and an estimated 375,000 deaths worldwide in 2020 [1]. The treatment management of PCa depends on the site and extent of disease (local/nodal vs. systemic disease) [2]. Even though several novel pharmacologic drugs have been introduced to the therapeutic armamentarium against metastatic PCa, advanced disease still represents a fatal condition for these patients [3]. For proper staging, imaging modalities such as CT, multiparametric MRI and bone scans are recommended in patients with intermediate risk and localized or locally advanced high-risk PCa [2]. In cases of biochemical recurrence (BCR) after radical prostatectomy or radiation therapy, functional PET–CT imaging using radiolabeled choline or prostate-specific membrane antigen (PSMA) ligands have been introduced [4]. PSMA is a transmembrane protein that is expressed in normal and neoplastic prostate tissue, with a structure composed of a 707-amino-acid external portion, a 19-amino-acid internal portion and a 24-amino-acid transmembrane portion [5]. In light of its specificity, PSMA has been selected as the biological target of a number of radiolabeled small molecules, such as [^68^Ga]-PSMA-11, [^18^F]-DCFPyL and [^18^F]-PSMA-1007 [6]. PSMA ligands can not only be coupled to diagnostic radionuclides such as [^68^Ga], [^18^F] and [^64^Cu], but also to therapeutic radionuclides (e.g., [^177^Lu], and [^225^Ac]), allowing a theranostic approach to PC diagnosis and treatment [7]. Previous studies have shown a high diagnostic performance of [^68^Ga]-labeled urea-based PSMA in the detection of lymph node metastases in BCR patients [8]. Additionally, PSMA ligands in molecular PET imaging provide a higher tumor detection rate as compared to choline ligands in patients with BCR, especially in cases of very low PSA levels [9]. 

PSMA-PET has become a highly accurate staging tool in multiple settings in clinical routine, although its exact uses in clinical practice remain to be determined. We use it in our department for staging treatment-naïve locally advanced disease as well as recurrent and metastatic disease. To overcome the logistical difficulties of obtaining a [^68^Ga] generator, [^64^Cu]-PSMA and [^18^F]-PSMA have been introduced. [^64^Cu] possesses a relatively long half-life (t1/2) of 12.7 h, allowing imaging of smaller molecules, larger, slower clearing proteins and nanoparticles. Uniquely, it decays by three processes, namely, positron (17.8%, Emax = 0.65 MeV), electron capture (43.8%) and beta decay (38.4%, Emax = 0.57 MeV), and can thus be used for both treatment and imaging [10]. Its lower positron range compared to the commonly used [^68^Ga] grants a better spatial resolution. The high diagnostic potential of [^64^Cu]-PSMA PET–CT imaging has been clinically investigated in the past, and different chelators to [^64^Cu] are used (11). DOTA and NODAGA chelators form stable complexes with Cu and have been clinically used [11]. Another PSMA ligand which was introduced recently is ^64^Cu-PSMA-BCH, which was shown to have a high stability in vivo with a lower uptake in the liver than ^64^Cu-PSMA-617 [12]. PSMA I&T labeled with ^64^Cu also showed the feasibility of PET imaging through in vitro and in vivo studies [13]. In patients with low PSA values, a better performance was observed for ^64^Cu-PSMA-617 PET/CT compared to ^18^F-choline PET/CT in restaging after BCR [14].

In contrast, [^18^F] has been well established as a diagnostic radionuclide due to its physical and nuclear characteristics: a high positron decay ratio (97%), a relatively short half-life (109.7 min) and low positron energy (Emax = 0.63 MeV) [15].

The aim of our study is to evaluate the uptake behavior of [^64^Cu]-DOTAGA-PSMA with DOTAGA as a possible stable chelator compared to [^18^F]-PSMA PET–CT in a routine clinical setting in PCa patients.

## 2. Methods

We selected retrospectively a total number of 100 patients (50 examined with [^64^Cu]-DOTAGA-PSMA and 50 with [^18^F]-PSMA PET–CT), who were consecutively examined in our department, with biochemical recurrence after radical prostatectomy or had progressive local disease in the last 3 months and were scheduled for either radiation or systemic therapy. Routinely, we perform, due to logistic reasons, [^64^Cu]-DOTAGA-PSMA one day per week and [^18^F]-PSMA PET–CT one day per week. All patients were examined with a dedicated PET–CT scanner (Biograph; Siemens Healthineers) in compliance with the 1964 Declaration of Helsinki and the responsible regulatory bodies in Austria. Formal consent was obtained from all patients prior to examination. A total of 250 MBq (3.5 MBq per kg bodyweight, range 230–290 MBq) of [^64^Cu]-DOTAGA PSMA or [^18^-F] PSMA was applied intravenously. PET images were performed 1 h post-injection (skull base to mid-thigh). The SUVmax of the suspected lesions and the SUVmean of the right liver lobe were measured. 

### Statistical Analysis

For the two patient cohorts the median values of age, PSA value at time of PET–CT, the SUVmean of the right liver lobe and the SUVmax of the lesion with the highest uptake as well as the total number of lesions are presented in Table 1.

## 3. Results

**Figure 1:**
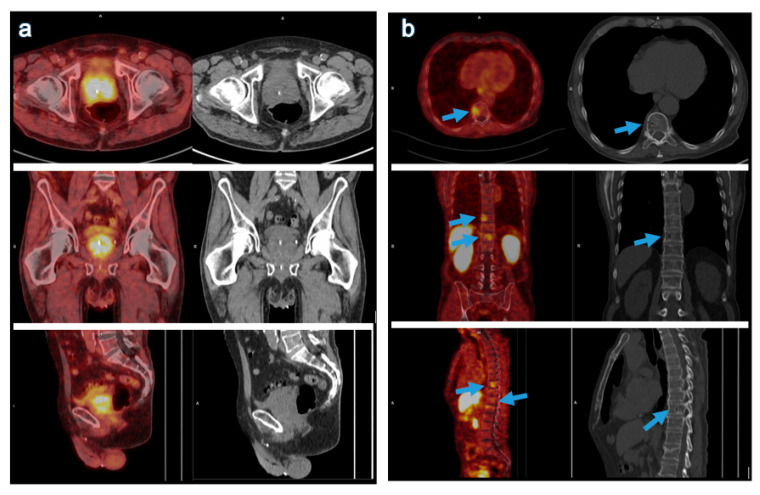
Primary staging of a treatment-naïve, 75-year-old patient with PCa which has metastasized to the bone, using [^64^Cu]-DOTAGA-PSMA PET–CT. The PSA value at the time of PET–CT was 47 µg/L. (**a**) The PSMA-positive primary tumor (SUVmax apical: 9.5; left base: 8.0). (**b**) The PSMA-positive bone metastases in T10 and T12 (respective SUVmax: 5.3 and 4.6); the arrow in the correlating low-dose CT images point to the metastasis in T10.

**Figure 2:**
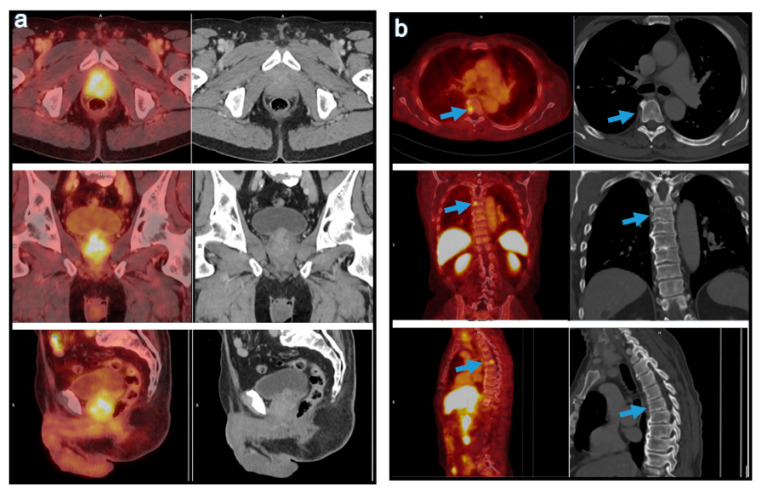
Primary staging of a treatment-naïve, 70-year-old patient with PCa metastasizing to the 5th and 9th thoracic vertebrae, using [^18^F]-PSMA PET–CT. The PSA value at the time of examination was 7.8 µg/L. (**a**) The PSMA-positive primary tumor (SUVmax 6.4). (**b**) The PSMA-positive bone metastasis in T5 (SUVmax 3.7).

Patient baseline characteristics are listed in Table 1. The two patient cohorts were comparable, as we found no significant differences with regard to age range, PSA values at the time of examination and the SUVmax of the hottest lesion (Table 2). In Figure 1 and Figure 2 we show one representable patient case for each cohort. 

In total, 209 lesions were detected with [^64^Cu]-DOTAGA-PSMA, whereas 191 lesions were with [^18^F]-PSMA. All but 9/50 (18%) of the patients (PSA range 0.01–0.7) studied with [^64^Cu]-DOTAGA PSMA and 6/50 (12%) of the ones (PSA range 0.01–4.2) studied with [^18^F]-PSMA had no lesions shown by PET–CT. We did not find significant differences in median PSA values between the two groups (*p* = 0.1) or any association between prostate uptake and the number of positive lesions in PSMA PET.

## 4. Discussion

The results in this study in regard to negative findings are superior to our previous study with [^64^Cu]-NODAG-PSMA, where 20.8% of the patients did not show any lesions in PET scans [11]. However, the previous study was performed on a stand-alone PET scan (without CT), and therefore this comparison is of limited significance.

The total number of lesions was higher with [^64^Cu]-DOTAGA-PSMA (209 vs. 191); however, the median value of lesions was one for [^64^Cu]-DOTAGA-PSMA and two for [^18^F]-PSMA, and thus failed to be statistically significant (*p* = 0.07). Interestingly, the median value of SUVmean of the right liver lobe was significantly higher with [^18^F]-PSMA (*p* < 0.05). We measure and mention this parameter in our PET reports in order to compare different studies at different time points, or if they are performed with different radiopharmaceuticals. 

In our study, we found comparable results between [^64^Cu]-DOTAGA-PSMA and [^18^F]-PSMA in our patients. The performance of both radiopharmaceuticals were comparable and better than our previously published results with [^64^Cu]-NODAGA-PSMA PET, with the limitation that the latter was performed on a stand-alone PET scanner [11]. This suggests in vivo stability of [^64^Cu]-DOTAGA-PSMA as it could be shown for [^64^Cu]-NODAGA-PSMA in our previous study [11]. Additionally, as mentioned elsewhere, [^64^Cu] as a radionuclide for PSMA shows more favorable physical characteristics, such as a longer half-life of 12.7 h and a small positron range with increased spatial resolution [16]. Interestingly, in a study, ^64^ Cu-PSMA-617-PET was demonstrated to be feasible for imaging prostate cancer for both the primary tumor site and metastases, whereas later imaging 2–22 h post-injection showed no additional, clinically relevant benefit compared to the early scans [17].

It was not the aim of this study to look for any impact of Gleason score (GS) on PET results; however, in a previous publication we could not find any correlation between GS and PSMA-PET [11]. Furthermore, downregulation of PSMA expression due to androgen deprivation therapy (ADT) might have an influence on size reduction in tumors [18], and some of our patients were under ADT treatment. Additionally, there are published limitations of [^68^Ga]-PSMA with a very low expression of PSMA in dedifferentiated tumors, the absence of a relationship between [^68^Ga]-PSMA uptake and Gleason score in addition to the downregulation of PSMA expression by ADT [18]. The introduction of copper as a ligand in primary staging and in recurrent disease demonstrates an excellent resolution of the detected lesions with a very high lesion-to-background contrast. Grubmüller et al. investigated the diagnostic potential of [^64^Cu]-PSMA-617 PET/CT in primary staging or as PSMA radioligand therapy in 29 PCa patients [19]. The preliminary results of this study highlighted the high potential of [^64^Cu]-PSMA ligands in patients with recurrent disease and in the primary staging of selected patients with progressive local disease. Our results with [^64^Cu]-DOTAGA-PSMA compared to [^18^F]-PSMA PET–CT demonstrate a similar high detection rate of recurrent disease and a high stability in vivo for [^64^Cu]-DOTAGA-PSMA.

The limitations of this study are the retrospective nature, lack of intra-individual comparison, that the histopathology of all lesions was not obtainable in our patients and that only the number of lesions were analyzed. However, PCa was proven by biopsy or histopathology.

## 5. Conclusions

[^64^Cu]-DOTAGA-PSMA and [^18^F]-PSMA have comparable detection rates for the assessment of residual disease in patients with recurrent or primary progressive PCa. The uptake in the liver is moderately different, and therefore the SUV of the lesions in both studies would not be comparable. 

## Figures and Tables

**Table 1:** Patient characteristic of the two groups. Ranges are given in parenthesis.

	Median Age in y	Median PSA in ng/dl	Median SUVmean of the Liver	Total Number of Lesions	Median SUVmax of the Hottest Lesions
[^64^Cu]-DOTAGA-PSMA	74 (51–90)	5.59 (0.01–969)	8.9 (5.5–14.9)	209 (0–15)	10.5 (1.8–65.0)
[^18^F]-PSMA	73 (57–92)	4.5 (0.01–220)	11.8 (1.8–21.2)	191 (0–15)	9.2 (3.3–109)

Additionally, we performed a Student’s t-test to identify significant differences between the two patient cohorts.

**Table 2:** Results of a Student’s t-test for outlining potential significant differences between the two patient cohorts that were either examined with [^64^Cu]-DOTAGA-PSMA PET–CT or [^18^F]-PSMA PET–CT.

	Age in y	Total Number of Lesions	PSA Value in ng/dl	SUVmax Hottest Lesion	SUVmean Liver
*p*-value	0.3	0.8	0.1	0.7	0.000002 *

Statistically significant *p*-values < 0.05 are marked with (*).

## Data Availability

The above-mentioned data were acquired from our in-house PET–CT center. No other publicly archived dataset was analyzed.
